# Stereoselective Multicomponent Reactions in the Synthesis or Transformations of Epoxides and Aziridines †

**DOI:** 10.3390/molecules24030630

**Published:** 2019-02-11

**Authors:** Allan Ribeiro da Silva, Deborah Araujo dos Santos, Marcio Weber Paixão, Arlene Gonçalves Corrêa

**Affiliations:** 1 Centre of Excellence for Research in Sustainable Chemistry, Department of Chemistry, Federal University of São Carlos, 13565-905 São Carlos, SP, Brazil; allan.silva.bsb@hotmail.com (A.R.d.S.); deborah.araujo89@gmail.com (D.A.d.S.); mwpaixao@ufscar.br (M.W.P.); 2 São Carlos Institute of Chemistry, University of São Paulo, 13563-120 São Carlos, SP, Brazil

**Keywords:** multicomponent reactions, epoxides, aziridine, asymmetric synthesis, green synthesis

## Abstract

Small ring heterocycles, such as epoxides and aziridines, are present in several natural products and are also highly versatile building blocks, frequently involved in the synthesis of numerous bioactive products and pharmaceuticals. Because of the potential for increased efficiency and selectivity, along with the advantages of environmentally benign synthetic procedures, multicomponent reactions (MCRs) have been explored in the synthesis and ring opening of these heterocyclic units. In this review, the recent advances in MCRs involving the synthesis and applications of epoxides and aziridines to the preparation of other heterocycles are discussed emphasizing the stereoselectivity of the reactions.

## 1. Introduction

Heterocyclic compounds are very important due to their biological and/or pharmacological properties. In particular, the small ring heterocycles, such as epoxides and aziridines, are present in several bioactive natural products [1]. Among the epoxides, the following examples of enzymatic inhibitors deserve mention: scyphostatin, which was isolated from a mycelial extract of *Trichopeziza mollissima* by Nara et al. in 1999, is a neutral sphingomyelinase inhibitor [2]; and E-64, a promiscuous irreversible cysteine protease inhibitor that is broadly reactive toward the papain family, was isolated from *Aspergillus japonicus* in 1978 by Hanada et al. [3]. Moreover, 3,4-epoxy-6,9-heneicosadiene was identified as a sex pheromone component of the eucalyptus brown-looper *Thyrinteina arnobia*, which is considered an important pest in Brazilian native plants [4].

**Figure 1:**
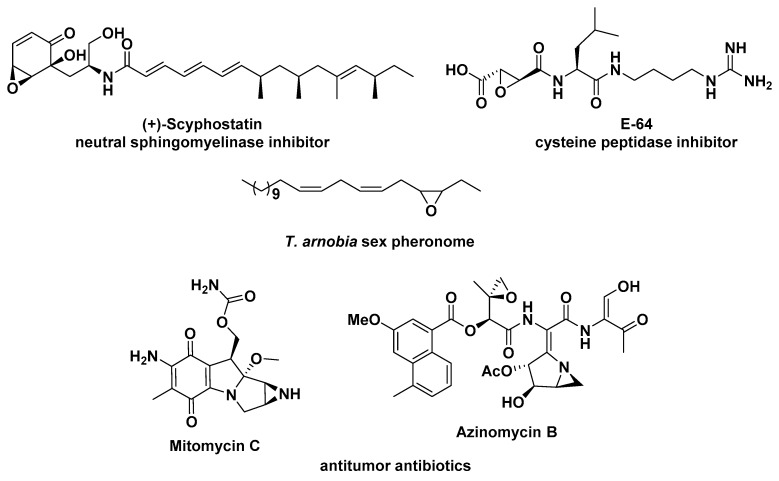
Examples of natural products possessing epoxide and aziridine rings.

Concerning the aziridine rings, we should emphasize the antitumor antibiotics mitomycin C, isolated from cultures of *Streptomyces caespitosus*, that has been used in combination chemotherapy for a variety of tumors and as an antifibrotic agent for several surgical procedures [5]; and azinomycin B, a remarkable natural product containing both epoxide and aziridine rings in its structure, originally isolated from *Streptomyces sahachiroi* (Figure 1) [6].

The three-membered rings are also highly versatile building blocks, frequently involved in the synthesis of natural compounds and incorporated into the molecular scaffold of numerous bioactive products and pharmaceuticals [7,8].

Several methodologies have been already reported for the stereoselective synthesis of epoxides and aziridines from alkenes catalyzed by transition metals, such as the seminal work by Professor Sharpless using Ti(O*i*Pr)_4_ and diethyl tartrate [9], the Jacobsen–Katsuki epoxidation employing Mn(III)–salen complexes [10,11], and also Rh(II)-aziridination [12,13]. The asymmetric synthesis of epoxides, cyclopropanes, and aziridines using organocatalysis was reviewed by Meninno and Lattanzi [14]. Moreover, a recent review covered the synthesis of natural and/or biologically relevant three-membered heterocycles and derivatives based on asymmetric epoxidation, aziridination, azirination, and thiirination reactions [15]. The use of three-membered rings as intermediates to the synthesis of biologically active compounds has also been extensively explored [16,17,18].

The development of efficient, stereoselective, and environmentally benign synthetic protocols has become extremely important in the last several decades. An effective approach to achieve these goals is to carry out a multistep reaction or synthesis in a one-pot procedure, since several synthetic transformations and bond-forming steps can be carried out in a single pot, while circumventing several purification procedures at the same time. This strategy, named “pot economy” by Professor Hayashi, minimizes chemical waste generation, saves time, and simplifies practical aspects [19].

An outstanding example by Smith III and Kim described the use of silyl 1,3-dithianes and epoxides in a solvent-controlled Brook rearrangement to access differentially protected monosilyl 1,5-diol moieties with precise stereocontrols [20]. This chemistry was further explored with the use of *N*-Ts aziridines, as the second electrophile to access protected 1,5-amino alcohols, which could be exploited as advanced intermediates for the construction of 3,5-disubstituted indolizidine rings. According to the authors, this transformation probably occurs via an intramolecular alkylation of the 1,5-amino alcohols, where the nitrogen would act as a nucleophile, attacking electrophilic carbons bearing activated oxygen substituents.

**Scheme 1:**
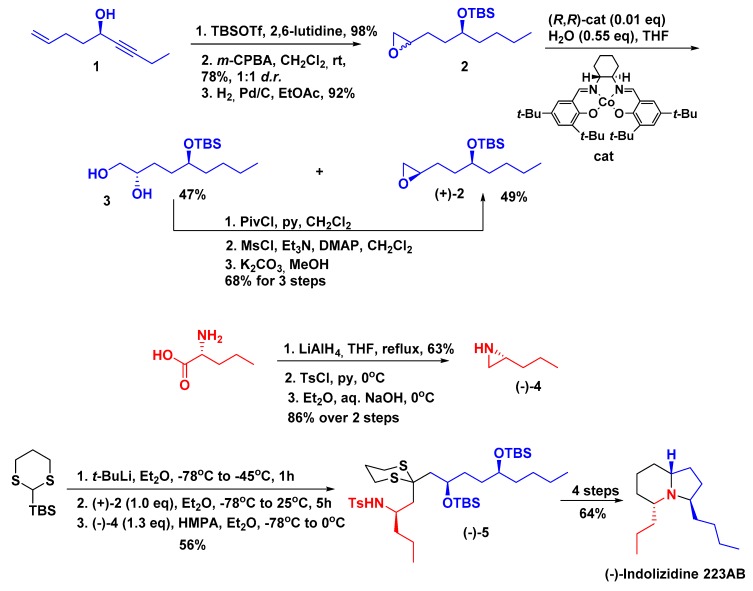
Total synthesis of (−)-indolizidine 223AB.

This strategy was applied in the total synthesis of (−)-indolizidine 223AB having epoxide (+)-**2** and aziridine (−)-**4** as building blocks (Scheme 1). The key step for the synthesis of chiral epoxide (+)-**2** involved a Jacobsen’s hydrolytic kinetic resolution (HKR) employing the (*R*,*R*)-Salen Co(III) catalyst [21]. Aziridine (−)-**4** was prepared from d-norvaline in three steps [22]. Then, lithiation of dithiane, followed by addition of epoxide (+)-**2**, warming to −25 °C over a period of 1 h, stirring for an additional 4 h at −25 °C, and then addition of aziridine (−)-**4** in Et_2_O containing hexamethylphosphoramide (HMPA, 0.65 equivalents) to promote the solvent-controlled Brook rearrangement furnished (−)-**5** in 56% isolated yield. Removal of the *t*-butylsilyl groups (TBS) using tetrabutylammonium fluoride (TBAF, 95% yield), bismesylation followed by treatment with potassium carbonate in MeOH for 3 h, and then addition of excess sodium amalgam (5%) directly to the reaction mixture furnished the desired product in excellent yield (95%). Finally, reductive removal of the dithiane with Raney Ni (69% yield) completed the synthesis of (−)-indolizidine 223AB.

The multicomponent and domino reactions have demonstrated a remarkable impact on the synthesis of complex products, with biologically or pharmacological active products among these [23]. The multicomponent reactions (MCRs) involve three or more reactants that are introduced concurrently and form a single product which contains the essential parts of the starting materials [24]. Such reactions allow the straightforward synthesis of intricate molecules in a one-pot fashion without the isolation and purification of intermediates, therefore leading to lower costs, time, and energy consumption. Additionally, MCRs are modular and convergent in nature and also an important source of molecular diversity [25]. Because of the potential for increased efficiency and selectivity, along with the advantages of environmentally benign synthetic procedures and catalyst reusability, association of heterogeneous catalysis and MCRs have been explored [26,27]. Recently, three-component reactions of amines, epoxides, and carbon dioxide have emerged as a powerful strategy for the synthesis of organic carbamates [28].

Despite the intensive research in the field of asymmetric synthesis in the last several decades, the asymmetric MCRs are still a challenge, especially those based on isocyanides (IMCRs) [29]. In this context, several groups have investigated asymmetric approaches to IMCRs, and recently very elegant solutions were found for the catalytic enantioselective version of some Passerini- or Ugi-type reactions [30,31]. Thus, in this review, the recent advances in MCRs involving the synthesis and applications of epoxides and aziridines is discussed with emphasis on the stereoselectivity of the reactions.

## 2. Epoxides

The epoxides are considered versatile starting materials in organic synthesis. The inherent polarity coupled with ring strain makes epoxides susceptible to various reactions with different reagents (nucleophiles, electrophiles, acids, and bases) to produce new useful functional groups.

### 2.1. Epoxide Synthesis

**Scheme 2:**
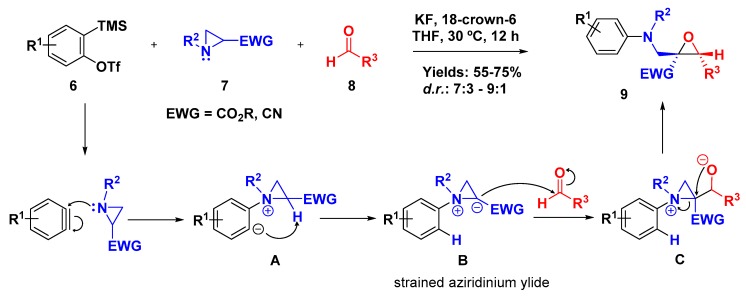
Three-component coupling involving aziridine, aryne, and aldehyde.

Amino epoxides are important building blocks in the synthesis of many natural products and analogues. Roy and coworkers demonstrated a transition-metal-free methodology for the synthesis of multisubstituted α-amino epoxides via a three-component reaction (3-CR), in which 2-(trimethylsilyl)aryl triflate (**6**) was employed to generate the aryne species in situ that reacted with *N*-substituted aziridines (**7**) and aldehydes (**8**) (Scheme 2). The authors proposed that the addition of aryne to the aziridine substituted with an electron withdrawing group (EWG) generated the zwitterionic species **A**; then, the intramolecular proton transfer provided a strained aziridine ylide **B** which added to the carbonyl component, and the resulting alkoxide **C** promoted the aziridine ring opening and further epoxide closure. This elegant approach furnished the desired epoxides in good yields and diastereoselectivity with good tolerance to different functional groups [32].

**Scheme 3:**
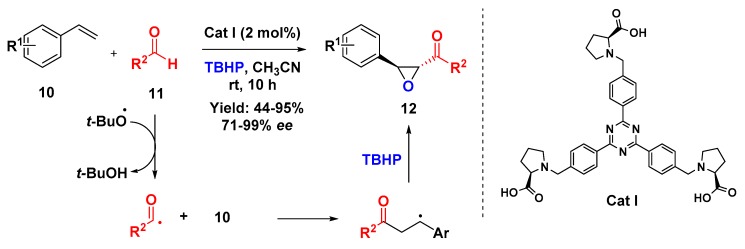
Proline-based chiral C_3_-symmetric organocatalyst for the synthesis of epoxide derivatives through a domino reaction.

Ashokkumar and Siva described a *C*_3_-symmetric proline-based organocatalyst (Cat I) for the synthesis of α,β-epoxy ketones (**12**) (Scheme 3) via a directly oxidative coupling involving styrenes (**10**) and aldehydes (**11**) in the presence of *tert*-butyl hydroperoxide (TBHP) as oxidant. The authors proposed that the reaction mechanism might follow a radical pathway in which the catalyst plays a crucial role to provide good enantioselectivity to the product. In general, the reaction presented a good tolerance for different substituents, including a less active aliphatic aldehyde [33].

**Scheme 4:**
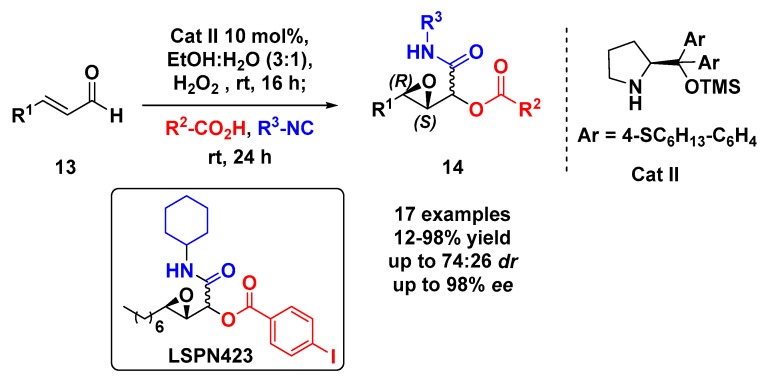
Tandem asymmetric organocatalytic epoxidation/Passerini reaction.

The use of asymmetric 3-substituted 2,3-epoxy-aldehydes in multicomponent reactions might be a challenge since those species can easily undergo epimerization under either acidic or basic conditions. To overcome this issue, our group designed the one-pot synthesis of epoxy-α-acyloxycarboxamides (**14**) from asymmetric organocatalyzed epoxidation of α,β-unsaturated aldehydes **13** followed by the Passerini 3-CR (Scheme 4). For the asymmetric epoxidation a new diarylprolinol silyl ether catalyst (Cat II) was developed, which enabled the use of green solvents such as ethanol/water mixture providing the desired epoxy-aldehydes with good yields and enantioselectivity. Then, the other components of Passerini-3CR (carboxylic acid and isocyanide) were added and after 24 h, highly functionalized epoxy α-acyloxycarboxamides **14** were obtained in good yields and stereoselectivity [34]. Those adducts were submitted to inhibition assays against cathepsins K, V, and L, in which the **LSPN423** was the most potent and selective against cathepsins L. Further investigations demonstrated that **LSPN423** is a tight binding uncompetitive inhibitor with an inhibition constant (K_i_) of 1.33 µM [35].

### 2.2. Epoxide Ring Opening

**Scheme 5:**
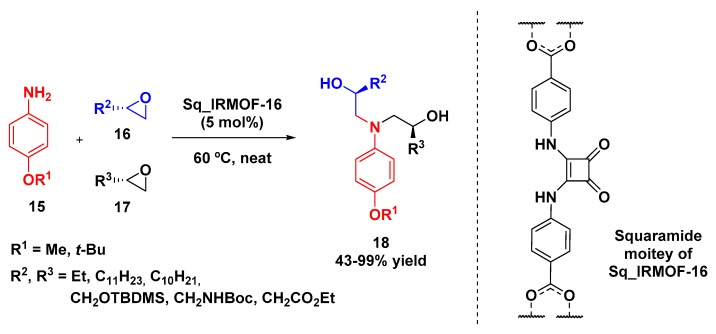
Epoxide ring-opening multicomponent reaction catalyzed by Sq_IRMOF-16.

Vignatti and Luis-Barrera recently described the synthesis of chiral 1,2,2′-aminodialcohols **18** via a three-component approach in which occurred the ring opening of two distinct epoxides **16** and **17** by a single aniline component (**15**) under solvent-free conditions. This was catalyzed by squaramide moieties present in a metal–organic framework (Sq_IRMOF-16) consisting of a zinc metal cluster (Zn_4_O) bridged by dicarboxylate linkers, forming a three-dimensional mesopore system (Scheme 5). The construction of the catalyst was planned so that the catalytic sites were available in all three dimensions, and also so that the pores were large enough to host the intermediates generated during the reaction. The authors demonstrated that MOF pores decorated with squaramide moieties were more effective than only the squaramide as catalyst in the proposed conditions. Enantiomerically pure epoxides were employed and the asymmetry was retained in the products. From the three-component reaction, they could obtain either homo-disubstituted (R^2^ = R^3^) or hetero-disubstituted amino diols (R^2^ ≠ R^3^) by controlling the addition of substrates. The polarity and size of the epoxide substituents directly affected the yield: bulky hydrophobic chains, such as –C_11_H_23_ and –C_10_H_21_, required longer reaction times (3–4 days) and provided the products in moderate yields (43 and 56%, respectively). On the other hand, an increase in substituent polarity lead to an increase of both rate and yield, even when bulky groups, such as –CH_2_OTBDMS, were used [36].

**Scheme 6:**
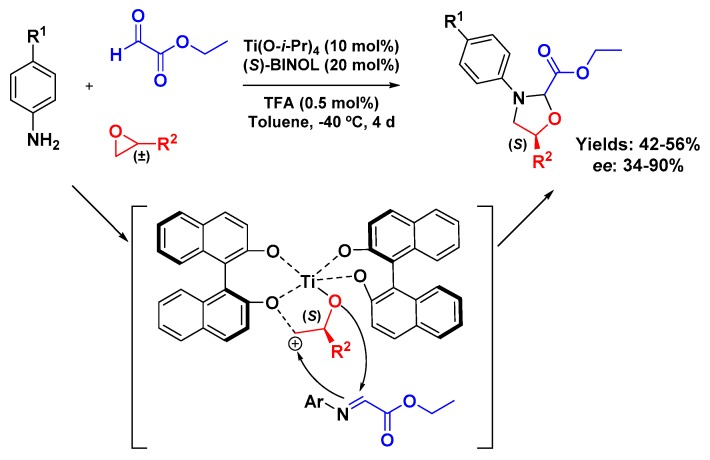
Asymmetric 3-CR and kinetic resolution of 1,3-oxazolidine derivatives.

The synthesis of multisubstituted asymmetric 1,3-oxazolidines **22** was reported by Hong et al. in a three-component reaction between anilines (**19**), mono-substituted epoxides (**21**), and ethyl glyoxalate (**20**) (Scheme 6). To obtain the enantiomerically enriched product, they used a Ti(IV) complex with the chiral ligand (*S*)-BINOL in a 1:2 ratio as catalyst, with a small amount of trifluoroacetic acid (TFA). Since only satisfactory yields were observed—around 50%—the authors further investigated the possibility that a kinetic resolution might be occurring.

Moreover, from the analysis of unreacted epoxide, they could detect that the substrate was no longer a racemate. Additional experiments have shown that only *S*-epoxide was consumed, confirming the kinetic resolution hypothesis. They also could observe the regiospecificity of the reaction, since from all the experiments—even at 10 mmol scale—only the C-4 isomer was detected; consequently, no C-5 isomer was found, which corroborates the proposed mechanism where the complex of Ti(IV) with (*S*)-BINOL promoted the epoxide ring opening (only *S*-isomer) that immediately reacts with the imine from aniline and ethyl glyoxalate, giving the desired product [37].

**Scheme 7:**
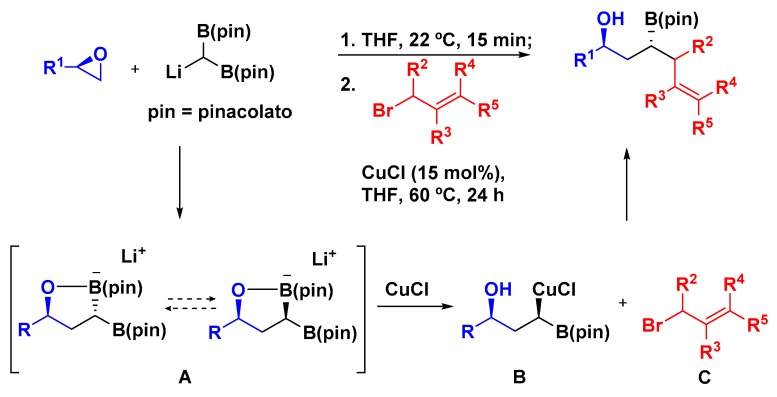
Copper-catalyzed three-component linchpin coupling.

Murray and coworkers described an anion relay strategy to the three-component linchpin coupling involving a tandem epoxide opening/Cu-catalyzed allylation, where two C–C bonds and one new stereogenic center were formed (Scheme 7). By using enantiopure epoxides **23** the authors observed excellent diastereoselectivity to the *anti*-isomer (up to 20:1). The [B(pin)]_2_–C(H)Li was employed as a bifunctional linchpin [38] so it could react with two electrophiles: first, promoting the epoxide opening with poor diastereoselectivity (intermediate **A**), and second, the subsequent deborylative transmetalation that generated the organocopper **B**, which reacted with the allyl bromide **C**, affording the desired products. According to mechanistic studies the step of formation of the organocopper species determines the *anti*-selectivity of the product. The 1,3-hydroxy-homoallylboronates **26** obtained were proven to be very useful for further functionalization, such as the stereoselective synthesis of a key intermediate of the alkaloid (+)-allo-sedamine; this methodology was also successfully extended to a sequential four-step process to obtain 1,3-polyol motifs [39].

**Scheme 8:**
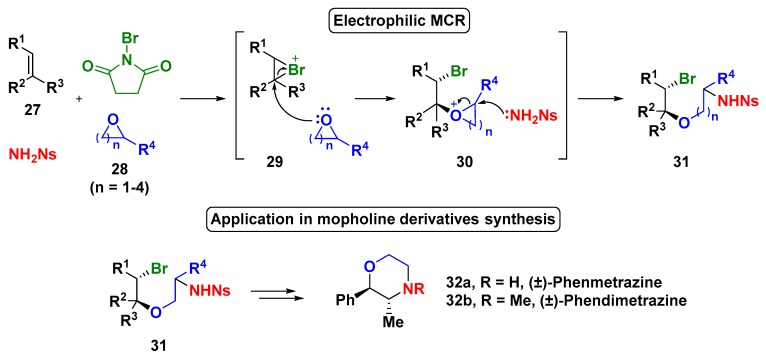
One-pot electrophilic aminoalkoxylation reaction.

In 2010, Yeung and coworkers first described a new catalyst-free electrophilic MCR involving olefins, cyclic ethers, primary amines, and *N*-bromo-succinimide (NBS) as a bromide source (Scheme 8) [40]. The initial studies used diverse cyclic ethers **28**. According to experimental observations, the authors proposed the MCR pathways in which NBS promoted the bromination of the olefin (**27**), then the cationic intermediate underwent ring opening by nucleophilic addition of the cyclic ether; in addition, the intermediate **30** was captured by the sulphonamide (NsNH_2_), furnishing the desired product **31**. The use of ethylene oxide as the cyclic ether component enabled subsequent reactions to provide biologically active morpholine derivatives, such as the (±)-phenmetrazine and (±)-phendimetrazine, two norepinephrine–dopamine releasing agents.

**Scheme 9:**
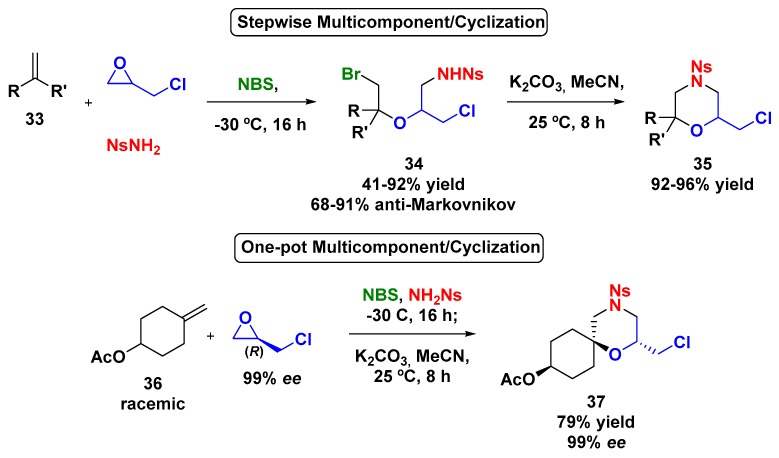
Synthesis of trisubstituted morpholines through an electrophilic multicomponent reaction using epichlorohydrin, olefin, nosyl amide, and *N*-bromosuccinimide.

In further investigations concerning electrophilic MCR employing epoxides, Zhou et al. observed that monosubstituted epoxides could be opened via Markovnikov or anti-Markovnikov pathways giving products with low regioselectivity (Scheme 9) [41]. Then, studies concerning the electronic effect of the epoxide substituents over the ring opening selectivity demonstrated a preference for the anti-Markovnikov product when epichlorohydrin was used. As previously described, the treatment of the multicomponent product **34** with K_2_CO_3_ in MeCN afforded the corresponding morpholines **35** in good yields. The attempt to perform both steps in one pot was successful, as well as the use of enantiopure (*R*)-epichlorohydrin in order to provide the asymmetric morpholine **37**; in this case, not only was the configuration of epoxide maintained, but the racemic olefin **36** was desymmetrized, affording a single stereoisomer with excellent enantiomeric excess.

**Scheme 10:**
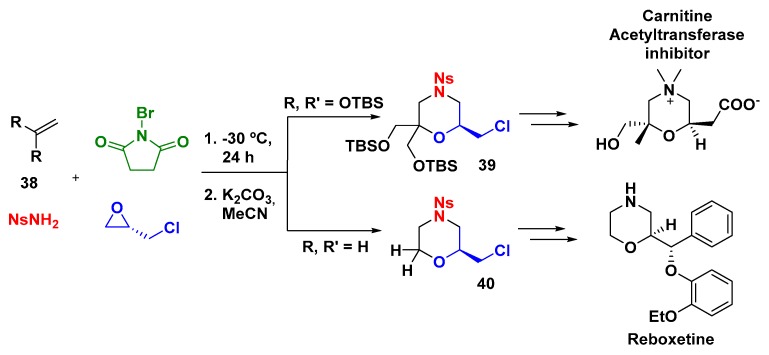
Formal synthesis of Carnitine acetyltransferase and Reboxetine.

In order to demonstrate the availability of the electrophilic MCR using epichlorohydrin in the synthesis of bioactive morpholines, Zhou and Yeung reported the formal synthesis of reboxetine, a norepinephrine reuptake inhibitor, and also the enantioselective synthesis of a carnitine acetyltransferase inhibitor (Scheme 10). The main strategy for the synthesis of both products built upon the previous methodology described by Yeung and employed tert-butyldimethylsilyl ethers (OTBS) or hydrogen as substituent to **38** aim to obtain the morpholine skeleton [41], followed by the substitution of chloride to introduce a suitable functionality, as well as removal of the nosyl group [42].

**Scheme 11:**
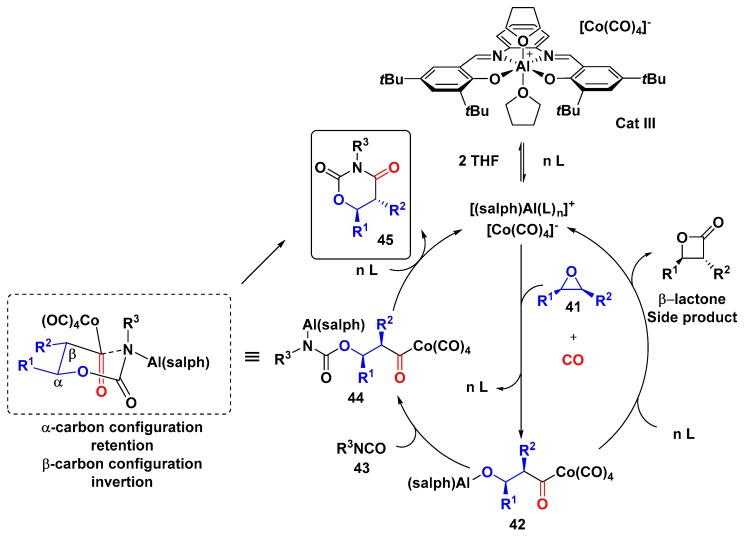
Three-component synthesis of 1,3-oxazinane-2,4-diones catalyzed by the [(salph)Al(THF)_2_]^+^[Co(CO)_4_]^−^ complex.

Coates et al. proposed a 3-CR with epoxides, carbon monoxide, and isocyanates based on previous mechanistic studies regarding the carbonylation of epoxides to form β-lactones catalyzed by the [(salph)Al(THF)_2_]^+^[Co(CO)_4_]^−^ complex, in which the intermediate **42** could be trapped by an electrophile such as isocyanates (Scheme 11). This 3-CR gave 1,3-oxazinane-2,4-diones (OD), a versatile intermediate in the synthesis of α,β-unsaturated carbonyl systems, β-ketoesters, and β-hydroxy-acid, among other functionalities. The substrate scope revealed that the electrophilicity of the isocyanate was crucial to the efficiency of the MCR, aryl isocyanates with electron-withdrawing substituents afforded the products with excellent yields and chemoselectivity; on the other hand, in the reaction with aryl isocyanates bearing electron-donating substituents, the products were obtained in lower yields and chemoselectivity, since β-lactones side products were observed. Therefore, the addition of intermediate **42** to isocyanate might be the rate-determining step. Further mechanistic studies gave additional information concerning reaction pathways: (1) β-lactone cannot be converted to OD; thus, β-lactone is not an intermediate in the OD synthesis; (2) NMR experiments with labeled OD prepared from ^13^CO confirmed the proposed mechanism, in which the CO was incorporated adjacent to the β-carbon; and (3) 1,2-disubstituted epoxides afforded OD with inversion of configuration in the β position to the ring oxygen and retention in the α position, e.g., *cis*-epoxide gave *trans*-OD and *trans*-epoxide gave *cis*-OD [43].

**Scheme 12:**
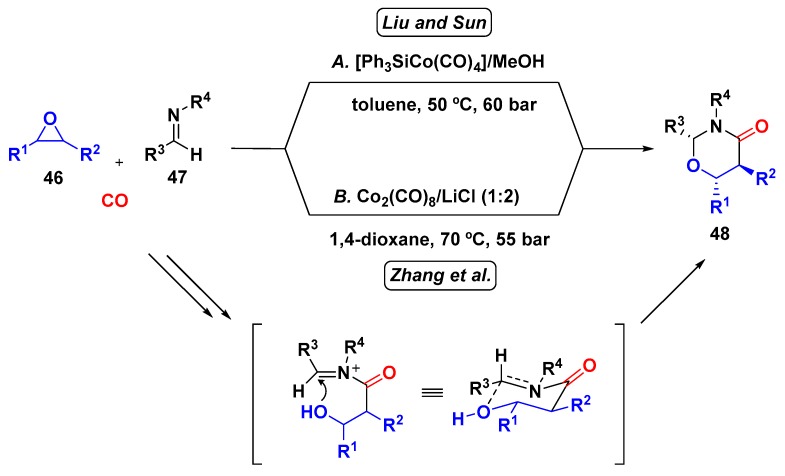
Cobalt-catalyzed 3-CR for the synthesis of 1,3-oxazinan-4-ones.

In 2014, Zhang et al. and Liu and Sun reported simultaneously a cobalt-catalyzed multicomponent reaction involving epoxides (**46**), imines (**47**), and carbon monoxide (Scheme 12) [44,45]. Interestingly, both groups aimed at the copolymerization of imines with CO, using the epoxide to generate acylcobalt species as a catalyst; however, the major product obtained was 1,3-oxazinan-4-ones (**48**), rather than the desired polypeptide. Since it was the first one-pot methodology described for the synthesis of this class of compounds, the authors further investigated this 3-CR. In general, alkyl-substituted epoxides provided the 2,6-disubstituted product due to the selective ring-opening at the less hindered carbon.

On the other hand, Liu and Sun observed the opposite regioselectivity for styrene oxide (2,5-disubstituted product), which was attributed to higher stability of the benzylic carbocation. The chairlike conformation of the six-membered cyclic transition state model can be used to rationalize the diastereoselectivity observed when monosubstituted epoxides were employed. They performed in situ IR experiments in order to obtain information concerning the reaction mechanism. The first account was that [Ph_3_SiCo(CO)_4_] pre-catalyst was converted to [HCo(CO)_4_]—the catalytic species—from β-hydrogen elimination of oxirane. To prove this hypothesis, they employed the [HCo(CO)_4_] generated in situ from the alcoholysis of [Ph_3_SiCo(CO)_4_] in methanol and the reaction proceeded at a higher rate. The IR experiments also indicated that imine addiction to the acylcobalt species is the rate-determining step of the reaction [40].

In the context of multicomponent reactions involving the transition metals, undoubtedly, the Catellani reaction is one of the most known. This reaction concerns an ipso functionalization of an aryl iodide and, concomitantly, a C–H activation in the ortho position catalyzed by palladium and promoted synergistically by 2-nobornene species. Recently, Zhang’s group developed an elegant method of C–H activation in a Catellani reaction employing epoxides, and, posteriorly, aziridine, as the alkylating agent for this transformation [46]. The salient features of the strategy include its broad substrate scope and its high atom economy, since an epoxide (or aziridine) can be incorporated into the product in its entirely, without the need for any sacrificial group.

**Scheme 13:**
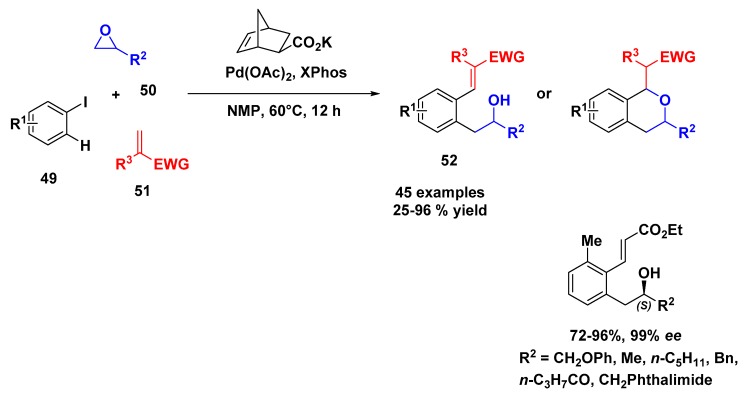
Palladium-catalyzed Catellani reaction using epoxides.

The developed protocol begins with the mixture of an aryl iodide **49**, an alkylating reagent (epoxide) **50**, and electron-deficient olefin as terminating reagent using Pd(OAc)_2_ as catalyst, XPhos as the ligand, the potassium salt of 5-norbornene-2-carboxylic acid as mediator, and *N*-methyl-2-pyrrolidone (NMP) as solvent at 60 °C (Scheme 13). After 12 h, the product was obtained in moderate to excellent yields (25–95%), and a total of 45 examples were prepared. Among these examples were the aryl iodide, the electron withdraw olefin, and a large range of epoxides including natural product derivatives and macrocyles. Chiral epoxides were also tested and a regioselective ring opening was observed for all products with no decrease of ee. Moreover, the products of the reaction are poised to undergo oxa-Michael reactions, thus allowing expedient access to isochroman scaffolds **52**.

## 3. Aziridines

Aziridines are a powerful synthetic building block widely used in the synthesis of different nitrogen-containing derivatives [47]. Due to their feasibility as synthetic precursors and building blocks, in this section we describe recent strategies for the aziridination and transformation of this heterocycle in multicomponent protocols.

### 3.1. Synthesis of Aziridines

There are many methods for the synthesis of aziridines and, currently, most of them involve diastereo- or enantioselective strategies to achieve this three-membered ring. However, few papers have reported the synthesis of this ring using three or more components.

**Scheme 14:**
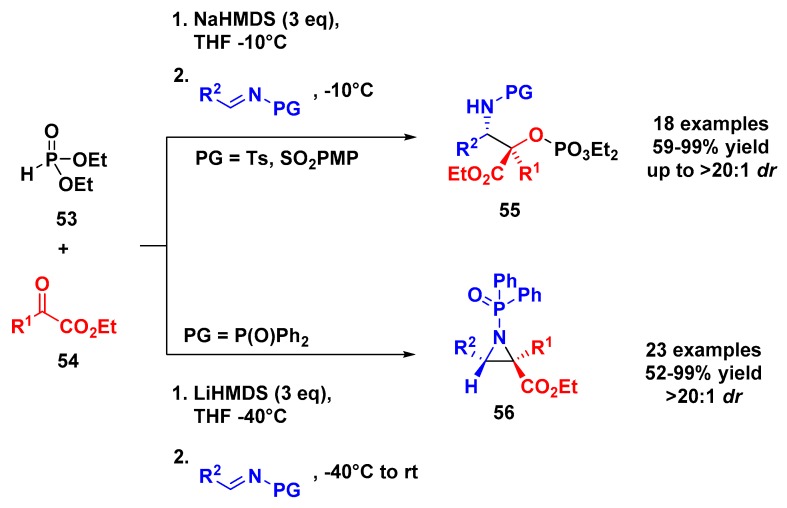
Three-component coupling of diethyl phosphite, α-ketoesters, and *N*-protected imines.

In 2016, Xu and coworkers described a three-component reaction for the preparation of α-phosphonyloxy-β-amino ester derivatives through an [1,2]-phospha-Brook rearrangement followed by addition of an imine substrate as an electrophile (Mannich coupling) (Scheme 14) [48]. In this MCR, using *N*-tosyl or *N*-4-methoxyphenylsulphonyl as a protecting group, 18 examples of α-phosphonyloxy-β-amino esters **55** were obtained in high yields and at >20:1 *dr* for most entries. Using diphenylphosphinyl as a protecting group, a different behavior was observed. This new protecting group increases the nucleophilicity of nitrogen, therefore facilitating the intramolecular elimination of the diethoxyphosphate group. This modification affords the aziridines **56** in good to excellent yields (52%–99%) and diastereoselectivity (>20:1 *dr*).

Concerning enantioselective protocols, aziridination from an MCR perspective has been scarcely described [49,50]. The first examples for this transformation were described in 1999 and consisted of stoichiometric reactions, whereas in 2009, organocatalyzed multicomponent aziridination reactions appeared in the literature [51,52]. Following Akiyama’s strategy [53], Bew and coworkers reported a multicomponent asymmetric Brønsted-acid-catalyzed aza-Darzens reaction for the synthesis of *N*-aryl-*cis*-aziridine carboxylate esters [54]. Alkyl diazo acetates and aromatic or hetero aromatic aldehydes were employed to afford 10 examples in good yields (61%–98%) and with mostly >90% ee; however, this score was dependent on the use of ortho-*tert*-butoxy aniline at −60°C to ensure the performance of this transformation.

**Scheme 15:**
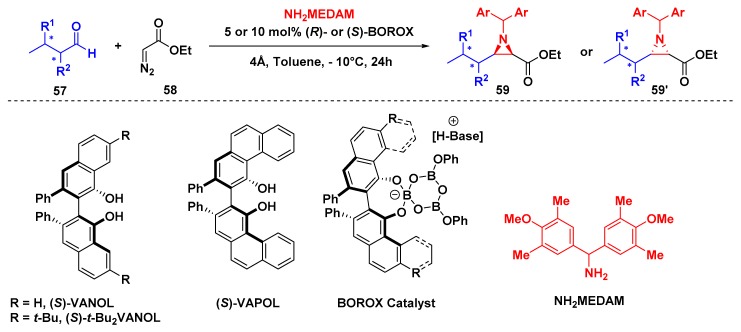
Multicomponent aziridination by BOROX catalysis.

Regardless of Bew’s reports, Wulff and coworkers had been contributing to the development of a multicomponent catalytic asymmetric aziridination reaction. The reported procedure starts with the addition of diazo ester compounds to *N*-protected aldimine catalyzed by a chiral Brønsted acid [55] (Scheme 15). This transformation usually affords *cis*-aziridine-2-carboxylate **59** in good yields and enantiomeric excess. This transformation begins with a facile method for in situ preparation of BOROX catalyst derivative from (*S*)-VAPOL or (*S*)-VANOL and three equivalents of B(OPh_3_). When the BOROX catalyst and protonated imine are close enough, an ion pair is formed and the diazo ester compound attacks, delivering the *cis*-aziridine.

Later, Wulff and coworkers evaluated the catalytic asymmetric *cis*-aziridination reaction from chiral aldehydes and the transition states using (*R*)- or (*S*)-BOROX. The reaction uses NH_2_MEDAM and α- or β-substituted chiral aldehydes at −10 °C and 5–10 mol % BOROX catalyst using (*R*)- or (*S*)-VAPOL, VANOL, and tBu_2_VANOL as ligands. A variety of different substrates reveal a strong dependence on the match between the substrate and the catalyst to achieve good yields and enantioselectivity.

**Scheme 16:**
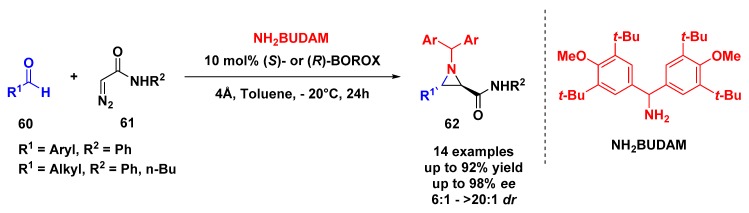
Enantioselective multicomponent *trans*-aziridination of imines with diazo acetamides.

Nevertheless, previous reports suggest that aziridination reactions using diazo acetamides instead of diazo esters afford *trans*-aziridine **62** [56,57,58]—the change in the stereochemical outcome could be rationalized by the reversal in the ordering of both the substrates **60** and **61** H-bonding to the BOROX catalyst, which causes a flip in the face selectivity of bond formation to the imine carbon [59]. Based on this information, Wulff and coworkers optimized the multicomponent *trans*-aziridination reaction for aryl and alkyl aldehydes **60** exploring the BUDAM protecting group on nitrogen (Scheme 16) [60]. With reaction conditions very similar to those employed before, the authors achieved excellent results for aromatic aldehydes, whereas alkyl aldehydes were highly dependent of the substrate, catalyst, and the time required for imine bond formation.

### 3.2. Aziridine Ring Opening

Over recent years, the aziridine moiety has been used in several methods involving ring opening strategies allowing access to domino chemical transformations in a one-pot procedure or without the purification/isolation of intermediates.

**Scheme 17:**
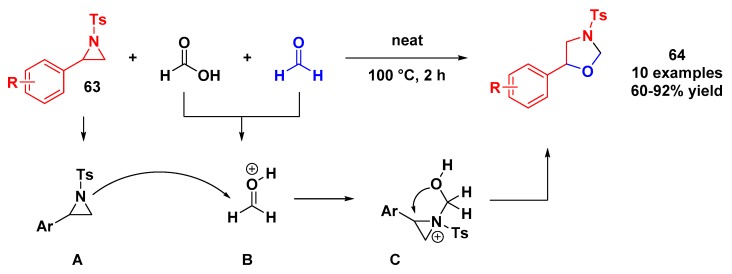
Protocol for the synthesis of 1,3-oxazolidines described by Majee and coworkers.

Based on the susceptibility of C–N bond cleavage in aziridine, Majee and coworkers reported the synthesis of 1,3-oxazolidines under neat conditions [61]. Based on previous work [62], the authors described a practical and simple multicomponent method to achieve this heterocycle. The protocol consists of a mixture of aziridine, formaldehyde, and formic acid in 1:1:1 ratio at 100 °C under neat conditions, and 1,3-oxazolidines were obtained in good yields (Scheme 17). The proposed mechanism begins with nucleophilic attack of nitrogen from the aziridine ring to the protonated formaldehyde. The oxygen atom adds to the benzylic position, opening the aziridine ring and closing the final five-membered oxazolidine.

**Scheme 18:**
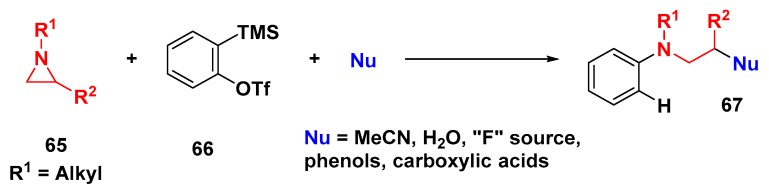
Aryne multicomponent coupling and use of aziridines as nucleophilic triggers.

Larionov and coworkers developed a 3-CR employing aziridine, aryne, and acetonitrile as the nucleophile [63]. Different research groups have since extended this concept to other nucleophiles such as fluoride [64], phenols, carboxylic acids [65], water [66], and aldehyde as the electrophile (Scheme 18) as shown before in Section 2.1.

**Scheme 19:**
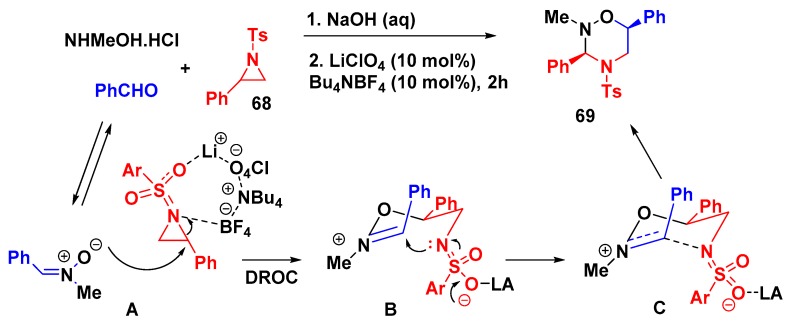
Multicomponent approach for the synthesis of 1,2,4-oxadiazinane.

Wani et al. developed a domino ring-opening cyclization (DROC) of activated aziridines and epoxides with nitrones via dual catalysis using LiClO_4_/Bu_4_NBF_4_ “on water” furnishing the corresponding 1,2,4-oxadiazinanes and 1,4,2-dioxazinanes, respectively. The authors also explored a model reaction in a multicomponent stepwise fashion, with benzaldehyde, *N*-methyl hydroxyl-amine hydrochloride, and 2-phenyl-*N*-tosylaziridine as substrates, obtaining the desired product 1,2,4-oxadiazinane **69** in good yield (78%) as a single diastereoisomer (Scheme 19) [67]. According to the proposed mechanism, the catalysts promoted the regioselective aziridine ring opening by nitrone followed by cyclization affording the *cis*-disubstituted product.

Another example of DROC of activated aziridines was published by Zhou and Yeung, who developed an electrophilic halogen-induced domino reaction for the synthesis of pyrrolidine, piperidine, or azepanes. The experimental protocol starts with an enantiopure olefinic aziridine, and *N*-bromosuccinimide (NBS) and NsNH_2_ as the halogenation agent and nucleophilic partner, respectively. For all cases, the aziridine **70** furnished good yields and stereoselectivity for the enantiopure substituted azepane, and no piperidine was detected [68].

**Scheme 20:**
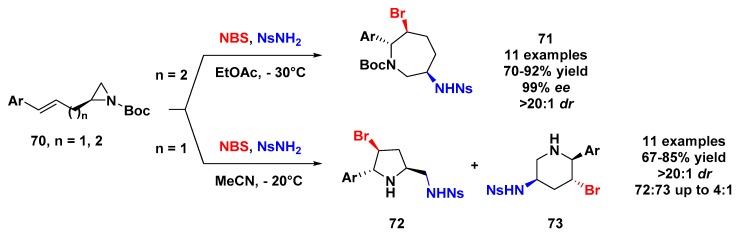
NBS-induced aminocyclization–aziridine ring expansion cascade of **70**.

From further investigations concerning electrophilic DROC employing ethenylaziridine (*n* = 1) [69], the same authors observed that an aziridinium ion intermediate formed in a shorter carbon chain could be opened by NH_2_Ns at either a terminal or internal position from aziridine to give pyrrolidine or piperidine, respectively (Scheme 20). The preference for the formation of pyrrolidine was attributed to the substitution having taken place at the less hindered terminal carbon position.

**Scheme 21:**
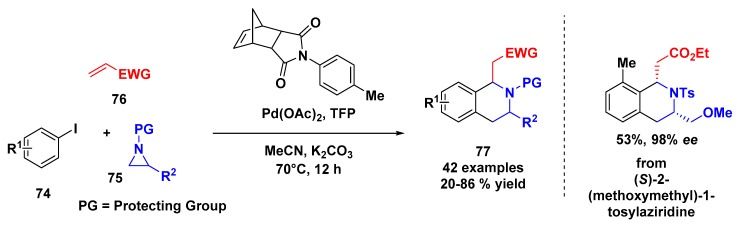
Palladium-catalyzed Catellani reaction for synthesis of tetrahydroisoquinolines.

Another example of aziridine opening reaction was described by Zhou and coworkers [70]. Inspired by the discovery that the epoxides could act as alkylating reagents for the Catellani reaction, the authors explored the same behavior for aziridine aiming for the synthesis of tetrahydroisoquinolines (Scheme 21). Using Pd/tri-2-furanylphosphine (Pd/TFP) as a catalyst, a norbornene mediator, K_2_CO_3_ in MeCN as solvent at 70 °C, the authors evaluated the aryl iodine, the *N*-protected aziridine, and electron-deficient olefin. Using this methodology, 42 examples were prepared in a mild, chemo- and regioselective ring opening, including an enantiopure aziridine. This protocol provided a versatile way to access tetrahydroisoquinolines.

**Scheme 22:**
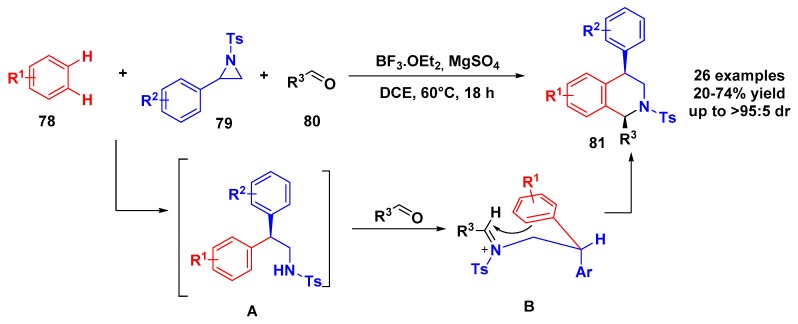
Tandem three-component reactions between aziridines, arenes, and aldehydes.

Beyond the last report, few papers are available on the one-pot multicomponent synthesis of tetrahydroisoquinolines. Based on the ready access and good reactivity of *N*-sulfonylaziridine, Xing and coworkers reported a stereoselective three-component synthesis of 1,4-disubstituted tetrahydroisoquinolines that provided a good choice for convergent synthesis of this core skeleton [71]. The 3-CR of aziridines, arenes, and aldehydes was performed with BF3OEt_2_ and anhydrous MgSO_4_ as an additive under dichloroethane (DCE) at 60 °C (Scheme 22). In most cases, *cis*-products were obtained in moderate yields with good regio- and diastereoselectivities. In the proposed mechanism, the Lewis acid promoted the ring opening of *N*-tosyl aziridine via a Fridel–Crafts substitution. Next, the imine sulfonyl compound further underwent Lewis-acid-catalyzed Pictet–Spengler condensation in a cascade fashion, leading to *cis*-1,4-disubstituted tetrahydroisoquinolines.

Yudin’s group expounded aziridine synthesis or functionalization, exploring the aziridine aldehyde dimer transformation in different ways, including diverse protocols involving MCRs. Enantiomeric enriched aziridine aldehyde dimers can be obtained from natural α-amino acids in five steps [72]. Recently, our research group described an alternative synthetic route to these dimers starting from α,β-unsaturated aldehydes through an asymmetric organocatalytic aziridination followed by Boc deprotection, under mild conditions and in two steps [73].

**Scheme 23:**
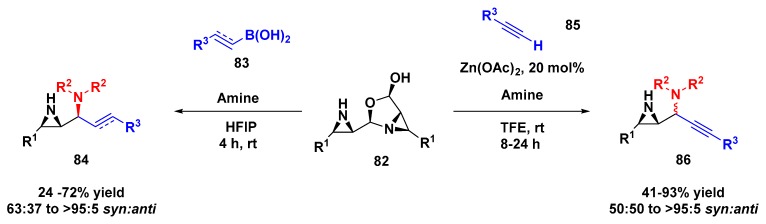
Example of aziridine aldehyde dimer as an inducing group for building blocks synthesis.

The aziridine aldehyde dimer has been employed as a building block to access, for example, a wide range of functionalized diamine compounds from a Petasis borono–Mannich process (Scheme 23). Using excess of morpholine, boronic acid, and aziridine aldehyde dimer under hexafluoroisopropanol (HFIP), the diamine **84** was obtained with high *syn* diastereoselectivity [74]. Another example was reported in 2017, in which the 1,2-aziridinyl propargylic amines (**86**) were obtained with good yield and stereoselectivity through a zinc-catalyzed multicomponent reaction under trifluoroethanol (TFE) at room temperature [75].

**Scheme 24:**
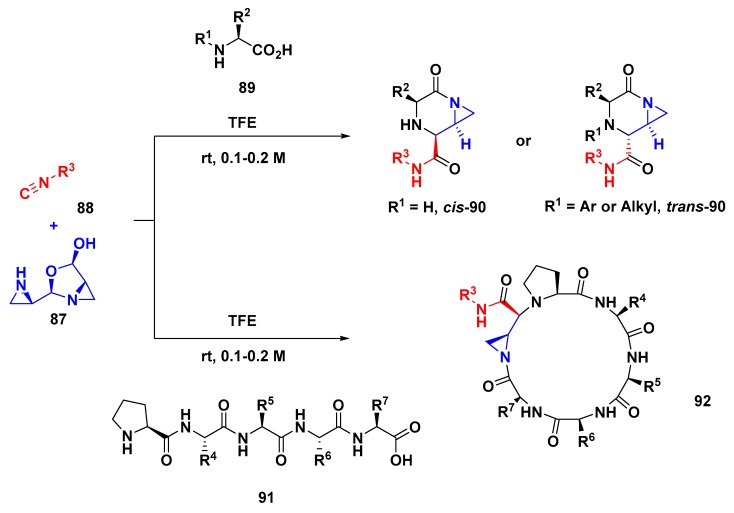
Disrupted Ugi reaction with aziridine aldehyde dimers, isocyanides, and amino acids (or peptides).

Furthermore, in 2010, Yudin’s group reported a *trans*-diastereoselective cyclization of amino acids and peptides using a disrupted Ugi reaction (as it was called by the authors) [76]. This protocol has as its main aspect the reversibly autoprotected aziridine aldehyde dimer **87** when applied in an Ugi reaction. This characteristic is related to iminium ion formation prior to the selectivity-determining isocyanide addition; then, the exocyclic aziridine intercepts the carbonyl group of the mixed anhydride, which undergoes solvolysis. The disrupted Ugi protocol afforded piperazinone **90** from three components; however, as showed in 2014 [77], there is a substantial difference in reactivity between secondary and primary amino acids in this kind of Ugi reaction (Scheme 24). The authors showed that the relative stereochemistry is controlled by both the amino acid and the aziridine aldehyde dimer under TFE. In the case of the chiral primary amino acid, this transformation was selective for the *trans*-substituted products **90** while the chiral secondary or protected amino acids afforded *cis*-products. Besides this, a diverse range of functionalized isocyanides were screened in the disrupted Ugi reaction to obtain chiral piperazinones in high stereoselectivities [78].

A computational study concerning the mechanism pathway, including the factors contributing to stereochemistry induction, was also reported by Yudin [79]. The same protocol was also extended subsequently to evaluate the multicomponent reactivity of linear peptides **91** towards peptide macrocycles **92** from disrupted Ugi reactions [80,81].

## 4. Conclusions

In conclusion, we have shown in this review that stereoselective MCRs can be efficiently employed in the synthesis of epoxides and aziridines, as well as in their transformation via ring opening on more functionalized compounds including other heterocycles, with potential pharmacological properties.

However, considering the green context of the MCRs, in several reports that were discussed in this work, quite toxic solvents were still employed, such as, for example, HMPA, DCE and THF. Although there is a long path to be travelled, alternative solvents that have low toxicity, are easy to recycle, and are inert should be considered. Furthermore, it is expected that investigations focusing on asymmetric catalysis will continue to grow in the near future, on the road to more sustainable chemistry.
